# Age-specific effects of synthetic cannabinoids on cognitive function and hippocampal gene expression in mice: insights from behavioral and molecular correlates

**DOI:** 10.3389/fphar.2025.1618929

**Published:** 2025-06-20

**Authors:** Kaixi Li, Yuanyuan Chen, Yawen Xu, Yuan Pang, Yongli Bao, Simeng Zhang, Xuesong Shi, Jingzhi Ran, Yanling Qiao, Yizhao Xu, Yiming Wang, Bin Di, Peng Xu

**Affiliations:** ^1^ School of Pharmacy, China Pharmaceutical University, Nanjing, China; ^2^ Office of China National Narcotics Control Commission, China Pharmaceutical University Joint Laboratory on Key Technologies of Narcotics Control, Beijing, China; ^3^ Department of Mechanical Engineering, Biomanufacturing Center, Tsinghua University, Beijing, China; ^4^ National Engineering Laboratory for Druggable Gene and Protein Screening Northeast Normal University, Changchun, China; ^5^ Key Laboratory of Drug Monitoring and Control, Drug Intelligence and Forensic Center, Ministry of Public Security, Beijing, China

**Keywords:** adolescent, synthetic cannabinoids, memory, transcriptomics, 4F-ABUTINACA

## Abstract

The increasing use of Synthetic cannabinoids (SCs) in adolescents and young adults poses significant medical and psychiatric risks, and previous reports have been dominated by single-age animal studies. Here, we first investigated the effects of a single exposure of the fourth-generation synthetic cannabinoid 4F-ABUTINACA on cognitive behaviors in adolescent (PND 28–35 days) and adult (PND 49–56 days) male mice in an animal model, followed by an age-specific systematic study by conducting a whole-gene transcriptomics study of hippocampal tissue in the brain. Behavioral results showed that 4F-ABUTINACA impaired recognition memory, fear memory extraction, and spatial navigation memory in adolescent mice, as well as spatial navigation memory in adult mice. The transcriptomics results revealed different alterations in age-enriched signaling pathways affected by 4F-ABUTINACA, such as Alzheimer’s disease, Parkinson’s disease, and neurodegenerative diseases. In addition, 4F-ABUTINACA causes selective downregulation of transcription of genes involved in stress response and mitochondrial expression in adolescent mice, whereas no significant differences were observed in adult mice. This study provides an innovative resource on the behavioral and molecular landscape of age-specific changes in cognitive function by synthetic cannabinoids and offers new opportunities for follow-up studies to target age-specific functional significance and related molecular mechanisms to be mined.

## Graphical Abstract

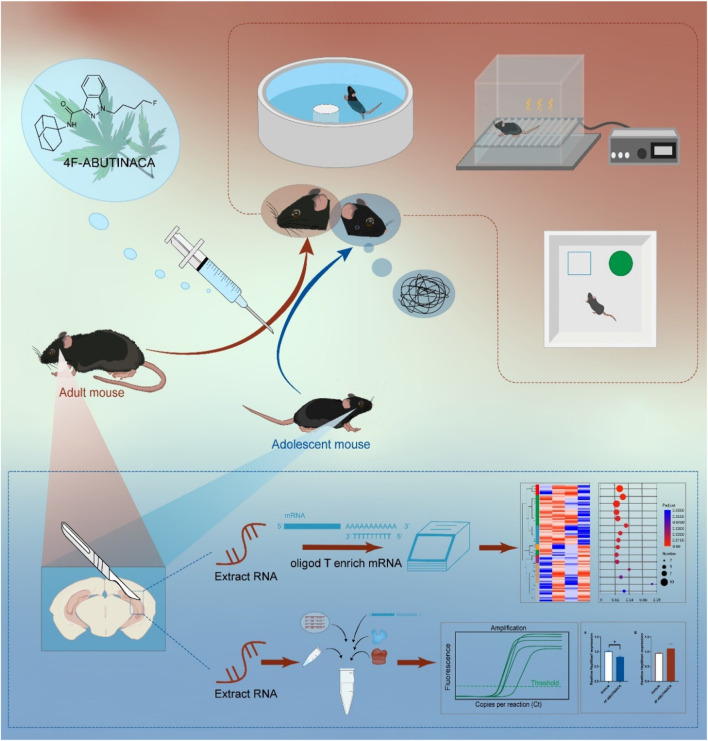

## 1 Introduction

Synthetic cannabinoids (SCBs) are a new class of psychoactive substances that act as potent full agonists at the cannabinoid receptors CB1 and CB2, with effects similar to those of the non-synthetic cannabinoid Δ9-tetrahydrocannabinol (Δ9-THC). However, unlike Δ9-THC, synthetic cannabinoids exhibit higher affinity and potent agonist properties, significantly higher than that of natural cannabinoid Δ9-THC (Ki∼10 nM) (Alsulaihim et al., 2025). Additionally, they can overactivate downstream signals: synthetic cannabinoids act as full agonists in Gαi protein activation and β-arrestin pathways (Patel et al., 2024), whereas Δ9-THC is only a partial agonist. Furthermore, they can cause unpredictable neurotoxicity and more severe organ damage than non-synthetic cannabinoids (Rcpsych, 2025). Synthetic cannabinoids have become a growing public health problem, primarily in Western societies (Davidson et al., 2017). Internet retailers and European ‘head shops’ promote SCB as meditative perfumes and tropical incense products under names such as K2 and Spice (Castaneto et al., 2014). These herbal mixtures, packaged in aluminum foil, often contain SCBs in a variety of structural classes, which are associated with greater adverse health effects than natural cannabinoids (Ford et al., 2017). For example, acute intoxication with SCBs has been associated with tachycardia, hypertension, visual and auditory hallucinations, mydriasis, agitation and anxiety, seizures, tachypnoea, nausea and vomiting (Davidson et al., 2017). Most alarmingly, abuse of SCBs can lead to death in some individuals (Trecki et al., 2015). Adolescents (Mathews et al., 2019) and military personnel (Santangelo et al., 2022) are the most common users, probably due to easy accessibility and the limited availability of selective and sensitive rapid analytical methods for screening these compounds (Bukke et al., 2021). When smoked by adolescents, SCs can produce significant psychiatric and physical effects similar to those of cannabis, such as altered cognition, behavioral disturbances, mood changes, and perceptual changes (Castellanos and Thornton, 2012) and the risk of developing psychiatric disorders is significantly increased. It has also been reported in the literature that young people (20–25 years old) driving a vehicle after using synthetic cannabinoid analogues have strong negative effects on the ability to drive a motor vehicle safely, with significant lane changing, inappropriate speed, collisions and severe inattentiveness (Louis et al., 2014), increasing the risk of becoming a danger to society.

Initially synthesized for research purposes, several first-generation SCRAs were diverted onto the NPS market at the beginning of the century. These preparations have been commonly sold as smokable herbal mixtures called “K2” (in North America), “Spice” (in Europe), “Youcatan”, “Chill”, or “Black Mamba”, and are allegedly safe for consumption (Adams et al., 2017). In 2008, for the first time, a recreational herbal mixture, allegedly containing a legal herbal blend mimicking cannabis relaxing effect, was found to contain the C8 homologue of CP 47,497 (2-[(1S,3R)-3-hydroxycyclohexyl]-5-(2-methyloctan-2-yl) phenol) and JWH-018 (Auwärter et al., 2009). By 2011, other SCRAs with different structures began to appear, and a second generation of synthetic cannabinoids based on the JWH-018 structure was synthesized. Examples include alkyl derivatives such as AM-2201. In early 2013, substances that fell outside the scope of the first and second generations of SCRA but subsequently entered the market and gained widespread acceptance constituted the third generation of SCRA. In early 2013, substances that fell outside the scope of the first and second generations of SCRA but subsequently entered the market and gained widespread use constituted the third generation of SCRA. such as 5F-ADB (methyl (S)-2-[1-(5-fluoropentyl)-1H-indole-3-carboxamido]-3,3-dimethylbutanoate) and MMB-2201 ((S)-methyl 2-(1-(5-fluoropentyl)-1H-indole- 3-carboxyamine)-3-methylbutanoate) in young people in Spain (aged 14–21) who consumed various herbal mixtures known as “spice,” “cherry bomb,” and “formula 6A” (Barceló et al., 2017). SCRAs remained the second most consumed NPS due to the emergence of the fourth generation of compounds, likely more potent and toxic than third-generation SCRAs. The current knowledge of the pharmacological and toxicological effects of this new generation shows that these compounds can cause serious harm to human health, although their mechanism of action is poorly understood (Malaca et al., 2022).

4F-ABUTINACA, also known as N-(4-fluorobutyl)-APINACA or 4F-ABINACA, is a fourth-generation synthetic cannabinoid (Malaca et al., 2022) with the IUPAC name N-(1-adamantyl)-1-(4-fluorobutyl) indazole-3-carboxamide. Its chemical structure closely resembles those of APINACA and 5F-APINACA (5F-AKB48), both of which are potent agonists of the CB1 and CB2 receptors (Canazza et al., 2016). Inclusion of a fluorine atom in the 4F-ABUTINACA structure, a halogen substitution that enhances lipophilicity and promotes blood-brain barrier permeation - a key determinant of CB1 receptor activation efficacy (Banister et al., 2015; Canazza et al., 2016). It is widely accepted that fluorination increases receptor binding affinity (2-5-fold potentiation) and the metabolic stability of psychoactive compounds (Gillis et al., 2015), which together explain the higher toxicity of 4F-ABUTINACA compared to non-fluorinated analogues (Banister et al., 2015). In our research group, as a joint laboratory focused on key technologies for the control of narcotic drugs, we have conducted preliminary studies on the safety and basic pharmacology of some fourth-generation synthetic cannabinoids. Our experimental results indicate that 4F-ABUTINACA exhibits higher acute toxicity, CB1 receptor affinity, and addictiveness compared to the first-generation synthetic cannabinoid JWH-018 (Luo, 2025). 4F-ABUTINACA has been identified in Asia in 2020 and fatal cases have been reported (Simon et al., 2023). Therefore, we have initiated more in-depth investigations into this compound.

As mentioned above, adolescents and young adults have the highest rates of use of SCBs (Mathews et al., 2019), which is a cause for particular concern. Adolescence is a sensitive period of neurodevelopment, during which exposure to cannabinoid-like substances can lead to sustained effects on multiple aspects of cognition (Scheyer et al., 2023). In contrast, most of the current studies on cognitive function alterations in SCs are based on a single age group, with adolescent or adult animal model studies, and age-specific systematic comparisons are at a gap stage. In this study, we will investigate the effects of a single administration of 4F-ABUTINACA on cognitive function in adolescence and adulthood, and reveal its molecular changes, so as to provide data support for subsequent mechanistic studies and targeted therapies for specific age groups.

## 2 Materials and methods

### 2.1 Animals

Adolescent C57BL/6 male mice (PND 28–35 days) and young adult C57BL/6 male mice (PND 49–56 days) (Spear, 2000)were obtained from SPF Biotechnology Co., Ltd (Beijing, China). All animals were housed in ventilated cages (4 per cage) with free access to chow and water in rooms maintained on a 12-h light/12-h dark cycle (lights on at 7:00 a.m., off at 7:00 p.m.) with constant temperature (25°C ± 2°C) and humidity (50% ± 10%). Mouse handling (weighing, operation, behavioral experiments, and sacrifice) was performed by female experimenters only, as suggested by Sorge et al. (2014). All experiments were conducted according to protocols approved by the Institutional Animal Care and Use Committee of China Pharmaceutical University (Approval No: KLDMC-WECLA-20240101).

### 2.2 Chemicals

4F-ABUTINACA was supplied by China Pharmaceutical University with a purity of 99%. It is first dissolved in 100% ethanol and then diluted to the desired volume with 5% Tween 80 and saline (0.9% NaCl). Solutions prepared with ethanol, Tween 80 and saline were also used as blank solvents. All drug solutions were freshly prepared before the experiments. All drugs were given by intraperitoneal (i.p.) injection. Mice were given 4F-ABUTINACA (1.2, 4 mg/kg, i.p.) or a vehicle 2 h before the tests, such as locomotor activity, Elevated Plus Maze (EPM), Novel Object Recognition (NOR), Contextual Fear Memory, and Morris Water Maze.

### 2.3 Locomotor activity

To investigate the effects of 4F-ABUTINACA on locomotor activity in mice, we measured the spontaneous activity of adolescence and adult mice for 15 min after administration. The locomotor activity chamber, consisting of an experimental box, a camera, a computer, and an automatic trajectory tracking and analysis system (Beijing Zongshi DiChuang Science and Technology Development Co., Ltd., Beijing, China), was used to record the distance of spontaneous locomotion and the average locomotor speed of the mice. Each mouse test chamber (35 cm × 35 cm × 35 cm) was an opaque Plexiglas box enclosed in a ventilated collection box (55 cm × 45 cm × 55 cm).

### 2.4 Elevated plus maze (EPM)

The EPM was adapted from (Alegre-Zurano et al., 2020) to assess anxiety-like behavior in mice. The apparatus (Beijing Zongshi DiChuang Science and Technology Development Co., Ltd., Beijing, China) consisted of a black maze with four arms (16 × 5 cm) arranged in the shape of a cross from a neutral central square (5 × 5 cm). Two arms were closed by vertical walls (closed arms), while the other two vertical arms had open edges (open arms). The maze was placed 30 cm above the floor. Camera, computer, and automatic tracking and analysis system (Beijing Zhongshi Dichao Technology Development Co., Ltd., Beijing, China) automatically tracked and identified the movement trajectories of mice in an elevated cross maze for 5 min, calculating the number of times they entered the open arm and the percentage of times they entered the open arm., and calculated the number of entries into the open arm and the percentage of entries into the open arm. The percentage of time spent in the open arms was calculated by dividing the time spent in the open arms by the sum of the time spent in the open and closed arms.

### 2.5 Novel object recognition test

The object recognition task was performed in a dimly lit test chamber (40 × 40 × 30 cm). Mice allowed to freely explore the test chamber for 15 min each day for the first 3 days of training to acclimatize to the test chamber. During training, two similar objects were placed symmetrically in the test chamber and each mouse was allowed to freely explore these objects for 15 min. Two hours after the end of training, a test was conducted in which one of the objects used in training (the familiar object) was replaced with a novel object. Each mouse was returned to the test room for 5 min, and the time spent exploring each object was recorded by a camera, computer, and an automated tracking and analysis system (Beijing ZhongshidiChuang Technology Development Co., Ltd., China). Exploration was defined as sniffing or touching an object with the nose and/or front paws, but climbing an object was not considered exploratory behavior. The discrimination index is used to assess memory retention and is calculated as (time spent exploring novel objects - time spent exploring familiar objects)/(total exploration time during the test).

### 2.6 Contextual fear memory

The conditioned fear system consists of a freeze-monitoring box, a soundproof box and a video fear conditioning system (Beijing ZhongshidiChuang Technology Development Co., Ltd., China). The freeze monitoring box (20 × 20 × 26 cm) is a transparent Plexiglas chamber with a removable door that contains a metal grid floor (0.3 cm grid spacing 0.8 cm) through which foot shocks can be delivered. The freeze-monitoring box was placed inside a soundproof box (42 × 46 × 52 cm), and the video fear conditioning system was mounted to the top of the soundproof box. Acoustic fear memory association learning was measured by the near-infrared video fear conditioning system. Training consisted of a single exposure to the situation (5 min), followed by paired learning of five tones [30 s, 5 kHz, 85 dB] and foot shocks (2 s, 0.6 mA, constant current). The mice were placed in the situation for 3 min during the test, and the sound was played for 30 s, after which the freezing time of the mice during the 30-s period was recorded as the result of the mice’s fear memory extraction.

### 2.7 Morris water maze

A round white pool (120 cm diameter, 40 cm height) with water (temperature 23°C ± 1°C, depth 35 cm) was used. The pool was divided into four quadrants with different cues outside the maze. A white escape platform with a diameter of 8 cm and a height of 20 cm was placed in one of the four quadrants. Spatial training trials were conducted for five consecutive days. Mice were trained four times per day in a randomized combination of the four quadrant training sequences, starting in one of the four quadrants. During each training session, mice were allowed to swim for 120 s to find the hidden platform (escape latency). If the platform was not found, the mice were guided to the platform and remained on it for 15 s before the next trial. Twenty-four hours after the last training trial, the mice were tested for memory. During the test, the platform was removed from the pool and the mice were allowed to swim for 60 s. During this time, an automatic video tracking system (Beijing ZhongshidiChuang Technology Development Co., Ltd., China) recorded the mice’s swimming trajectory, time spent finding the target platform, and swimming speed.

### 2.8 RNA sequencing (RNA-seq)

Mice were administered 4F-ABUTINACA (4 mg/kg, i.p.) or vehicle via intraperitoneal injection 2 h prior to the start of the experiment, after which hippocampal tissue was collected from the mice (the technique was repeated twice as a parallel group, with 3 parallel groups in each group) and extracted the total RNA using a Trizol kit (MJZol total RNA extraction kit, Shanghai Meiji Bio-pharmaceutical Science and Technology Co.). The RNA samples were submitted to the Majorbio (Shanghai, China). Then RNA quality was determined by 2100 Bioanalyser (Agilent Technologies, Santa Clara, CA, United States) and quantified using the ND-2000 (NanoDrop Technologies, Wilmington, DE, United States). Only high-quality RNA sample was used to construct the sequencing library. RNA-seq transcriptome library was prepared following the TruSeq™ RNA sample preparation Kit from Illumina (San Diego, CA, United States) using 1 μg of total RNA. After quantified by TBS380, the paired-end RNA-seq sequencing library was sequenced with the Illumina HiSeq xten/Nova Seq 6000 sequencer (2 × 150 bp read length).

### 2.9 Quantitative real-time polymerase chain reaction (qPCR)

Mice were injected with 4F-ABUTINACA (4 mg/kg, i. p.) or vehicle 2 h before the start of the test, and total RNA was extracted from the hippocampus tissue. Total RNA was extracted from the mouse hippocampus using a commercial RNA extraction kit (RNAfast200, Fastagen, Shanghai, China), and its concentration was quantified via a NanoDrop spectrophotometer (TermoFisher Scientific, Darmstadt, Germany). Quantitative RT-PCR was performed using 1 μg RNA and was converted to cDNA using the AdvanceFast first Strand cDNA Synthesis SuperMix Kit (YEASEN, Shanghai, China). Expression of mRNA was quantified by qRT-PCR using the CXF96TM Real-Time PCR system (BioRad, Hercules, United States), using a SYBR GREEN I Master Mix (11184ES08, YEASEN, Shanghai, China) GAPDH mRNA was used as an endogenous control (Primer sequences are available in the Supplementary Material). The reactions were performed in triplicate using 2 μL of cDNA in a 25-μL reaction volume. Specific cDNAs were quantified relative to a “calibrator” control sample serving as a reference. N = 3 mice/group.

### 2.10 Experimental design

We summarized the contents of the experiment in a simple table to more clearly describe the methods used in each test with the mouse group, as shown in Table 1.

**TABLE 1:** Schematic diagram of experimental design.

Experiment	Time window (Bialuk and Winnicka, 2011)	Adolescent mouse group	Adult mouse group	Sample size
Locomotor activity	Intraperitoneal injection (i.p.) 2 h before the start of the test	Vehicle,4F-ABUTINACA (1, 2, 4 mg/kg)	Vehicle,4F-ABUTINACA (1, 2, 4 mg/kg)	n = 7–8/group
Elevated Plus Maze	Intraperitoneal injection (i.p.) 2 h before the start of the test	Vehicle,4F-ABUTINACA (1, 2, 4 mg/kg)	Vehicle,4F-ABUTINACA (1, 2, 4 mg/kg)	n = 7–8/group
Novel object recognition test	Intraperitoneal injection (i.p.) 2 h before the start of the test	Vehicle,4F-ABUTINACA (1, 2, 4 mg/kg)	Vehicle,4F-ABUTINACA (1, 2, 4 mg/kg)	n = 7–10/group
Contextual fear memory	Intraperitoneal injection (i.p.) 2 h before the start of the test	Vehicle,4F-ABUTINACA (1, 2, 4 mg/kg)	Vehicle,4F-ABUTINACA (1, 2, 4 mg/kg)	n = 7–8/group
Morris water maze	Intraperitoneal injection (i.p.) 2 h before the start of the test)	Vehicle,4F-ABUTINACA (1, 2, 4 mg/kg)	Vehicle,4F-ABUTINACA (1, 2, 4 mg/kg)	n = 7–8/group
RNA sequencing (RNA-seq)	Intraperitoneal injection (i.p.) 2 h before the start of the test	Vehicle,4F-ABUTINACA (4 mg/kg)	Vehicle,4F-ABUTINACA (4 mg/kg)	n = 3/group
Quantitative Real-time Polymerase Chain Reaction (qPCR)	Intraperitoneal injection (i.p.) 2 h before the start of the test	Vehicle,4F-ABUTINACA (4 mg/kg)	Vehicle,4F-ABUTINACA (4 mg/kg)	n = 3/group

### 2.11 Statistical analyses

Results were presented as mean ± SEM. Data were analyzed using GraphPad Prism 9 (GraphPad Software, Inc., La Jolla, CA, United States). Statistical analysis was conducted using Student’s t-test, one-way ANOVA followed by HolmSídak’s multiple comparisons test, or two-way ANOVA followed by Sídak’s multiple comparisons test. Significance was shown as *P < 0.05, **P < 0.01, ***P < 0.001, or n. s (not significant).

## 3 Results

### 3.1 Effects of 4F-ABUTINACA-induced locomotor activity and anxiety-like behavior

**FIGURE 1:**
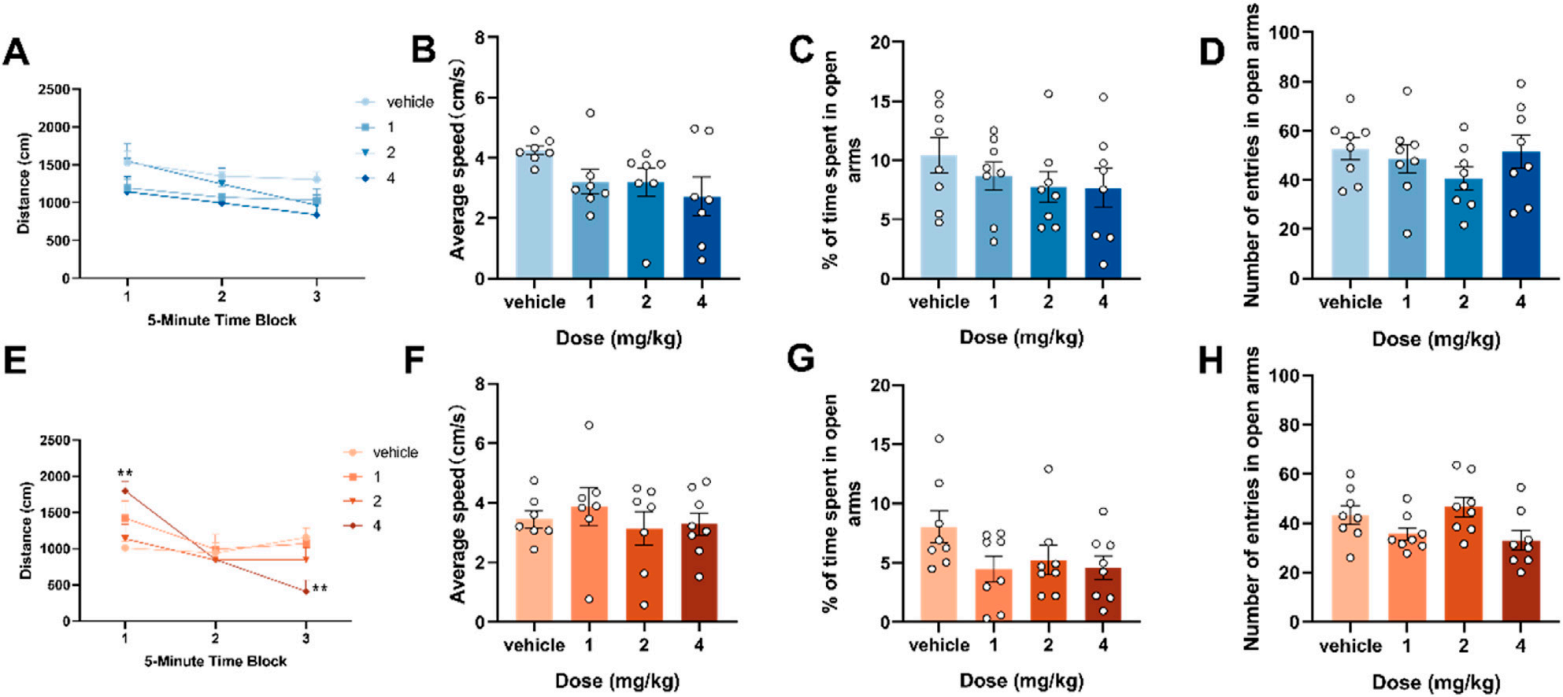
Effects of adolescent and adult exposure to 4F-ABUTINCA on locomotor activity and anxiety-like behavior. Effects of exposure to 1, 2, and 4 mg/kg of 4F-ABUTINACA in adolescent **(A–D)** and adult **(E–H)** mice on the distance between consecutive 5-min intervals, average speed, time spent in the open arm, and number of open-arm entries. n = 7**–**8 mice per group, Data are expressed as mean ± SEM. *P < 0.05, **P < 0.01 (comparison between 4F-ABUTINACA and vehicle group; two-way ANOVA with repeated measures, treatment **(A,E)**; one-way ANOVA **(B–D)**, **(F–H)**.

**FIGURE 2:**
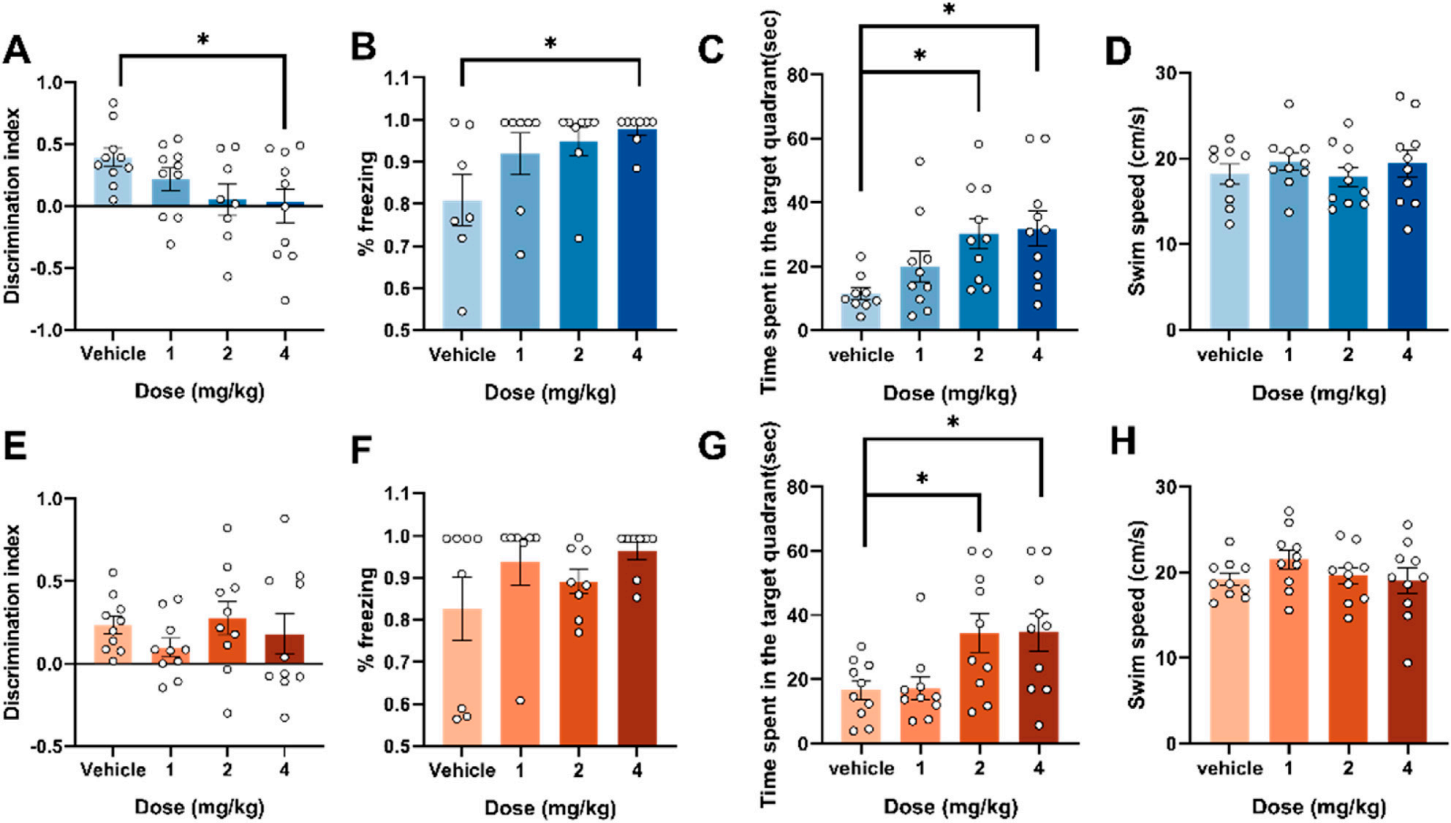
Effects of adolescent and adult exposure to 4F-ABUTINCA on recognition memory, fear memory and spatial memory. Effects of exposure to 1, 2, and 4 mg/kg of 4F-ABUTINACA in adolescent **(A–D)** and adult **(E–H)** mice on the discrimination index, %freezing, time spent in the target quadrant and swim speed. n = 7–10 mice per group, Data are expressed as mean ± SEM. *P < 0.05, (comparison between 4F-ABUTINACA and vehicle group; one-way ANOVA).

Based on previous experimental results (Luo et al., 2025), the median effective dose (ED_50_ value) that caused motor inhibition in mice in an open field test 30 min after intraperitoneal injection in ICR mice was 4.65 mg/kg. Our experimental dose selection was based on this data, we measured spontaneous activity and anxiety-like behavior in adolescent and adult mouse 2 h after intraperitoneal injections of 1, 2 and 4 mg/kg of 4F-ABUTINACA to explore the locomotor ability and anxiety would affect the results of subsequent experiments on tests related to memory ability. The results of the experiments showed that adolescent mice did not show statistically significant differences in mean locomotion and distance of locomotion compared to the vehicle group 2 h after injection (Figures 1A,B), and adult mice showed statistically significant differences in distance of locomotion compared to the vehicle group at 4 mg/kg (F (6,72) = 3.589, P = 0.0036) (Figure 2E), while the mean speed of locomotion did not have a statistically significant effect on locomotion compared to the vehicle group (Figure 1F). Tracking plot to observe the differences between vehicle, 1, 2, and 4 mg/kg of 4F-ABUTINACA mice, with specific results shown in Supplementary Figure S1. In the elevated plus maze test, the results showed that in both adolescent and adult mice, the time spent in the open arm (Figures 1C,G) and the number of times to enter the open arm (Figures 1D,H) were not statistically different compared with the vehicle group.

### 3.2 Behavioral investigation of the effects of 4F-ABUTINACA on recognition memory, fear memory and spatial memory in adolescent and adult mice

We then examined the differences in memory behavioral performance between adolescent and adult mice 2 h after injection in a novel object recognition experiment, a conditioned fear memory experiment and a morris water maze experiment, and the results are shown in Figure 2. In the novel object recognition test, adolescent mice exhibited a significant reduction in the discrimination index at a dose of 4 mg/kg (F (3,34) = 2.693, P = 0.0615) (Figure 2A). In the fear memory retrieval test, adolescent mice also exhibited memory retrieval impairments at the same dose—increased freezing time (F (3,26) = 3.037, P = 0.047) (Figure 2B). In contrast, adult mice showed no statistically significant differences compared to the control group in both experiments (Figures 2E,F). In the Morris water maze experiment, adolescent and adult mice exhibited similar spatial memory deficits, characterized by significantly increased time spent in the target quadrants. Two hours after intraperitoneal injection of 4 mg/kg 4F-ABUTINACA, adolescent mice (F (3,35) = 4.188, P = 0.0124) and adult mice (F (3,36) = 4.513, P = 0.0087) showed statistically significant differences compared to the control group (Figures 2C,G). We also measured swimming speed, which showed no statistically significant differences compared to the control group, indicating that the effects of 4F-ABUTINACA on spatial memory ability are independent of motor ability (Figures 2D,H). A tracking trajectory plot is also provided to observe the differences between mice treated with vehicle, 1 mg/kg, 2 mg/kg, and 4 mg/kg of 4F-ABUTINACA. The specific results are shown in Supplementary Figure S2.

### 3.3 4F-ABUTINACA-induced effects on hippocampal transcription in the brain: overall effects

**FIGURE 3:**
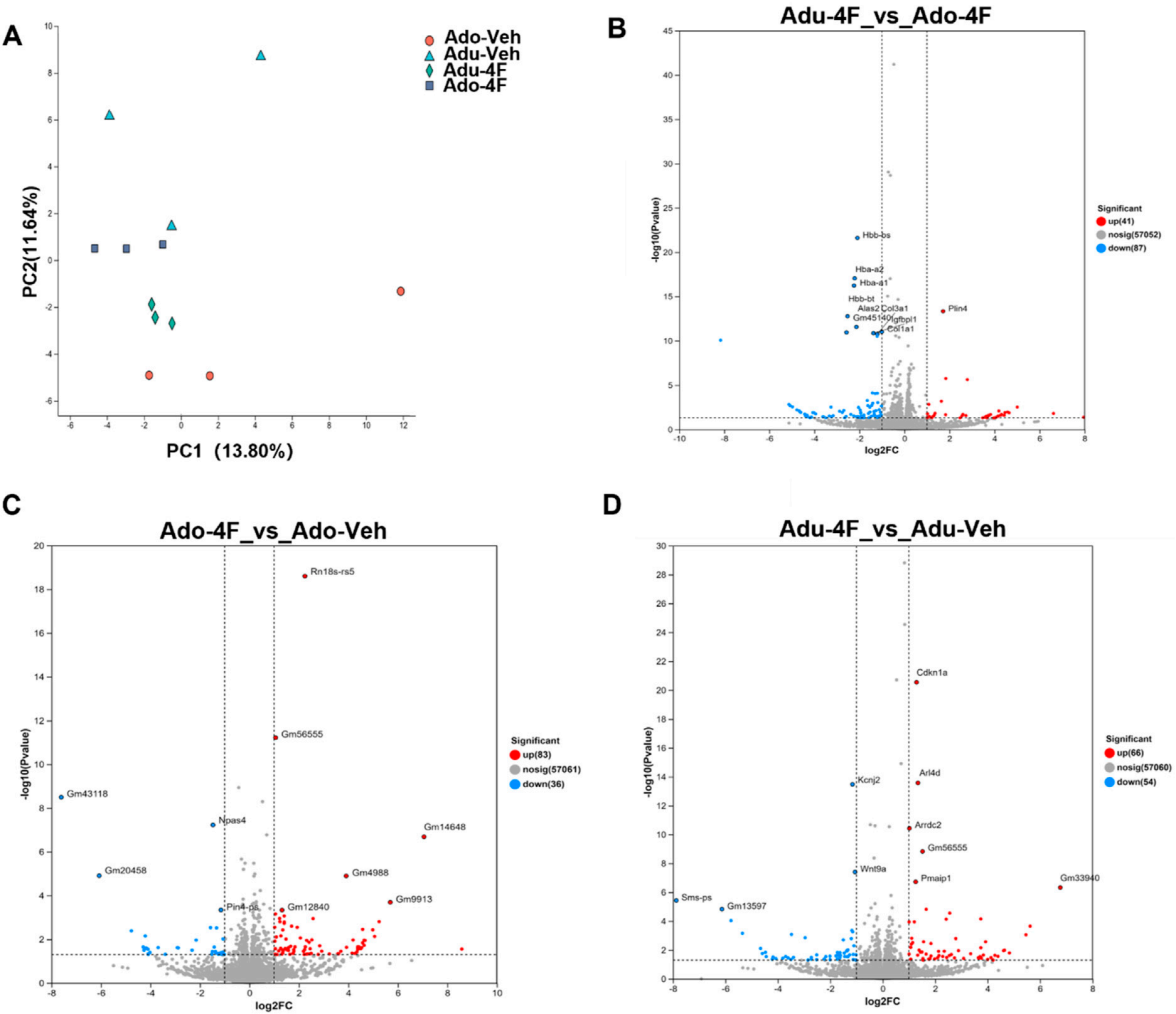
Effects of adolescent and adult exposure to 4F-ABUTINCA overall effects. principal component analysis of RNAseq data on hippocampal tissues of adolescent and adult mice after exposure to 4F-ABUTINCA (4 mg/kg, i.p.) or vehicle control **(A)**. Volcano plots of differentially expressed genes (Padj <0.05) in adolescent and adult mice after 2 h exposure to 4F-ABUTINCA (4 mg/kg, i.p.) **(B)** or vehicle **(C,D)**. Statistical analyses were performed as described in 2.8. RNA sequencing and bioinformatics analyses are described in the “Materials and Methods” section.

Next, we examined the effects of adolescent and adult 4F-ABUTINACA (Ado-4F, Adu-4F) exposure on gene transcription in hippocampal tissue in the brain. We injected mice with 4F-ABUTINACA or its vehicle (Ado-Veh, Adu-veh), collected their hippocampi 2 h later and assessed transcription using bulk RNAseq. Principal component analysis (PCA) showed that gene transcription was significantly affected in both adolescent and adult animals (Figure 3A). Exposure of adolescent mice to 4F-ABUTINACA caused 128 genes to be differentially expressed (41 up; 87 down; Figure 3B) compared to adult mice, and in adolescent mice, 4F-ABUTINACA caused 120 genes to be differentially expressed (83 up; 36 down) relative to vehicle control (Figure 3C). In adult mice, 4F-ABUTINACA produced a similar effect, with 119 genes altered (66 up; 54 down, Figure 3D) after administration of 4F-ABUTINACA relative to vehicle control.

### 3.4 4F-ABUTINACA-induced effects on hippocampal transcription in the brain: differential gene function enrichment analysis

**FIGURE 4:**
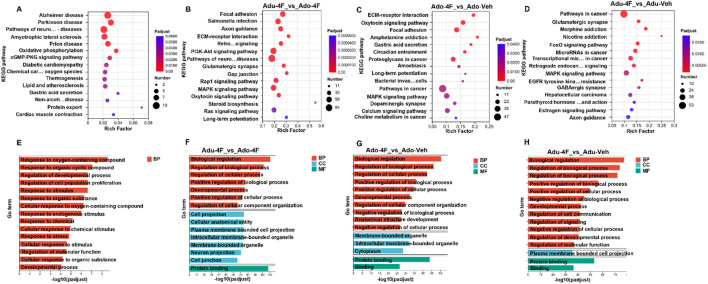
KEGG and GO analyses of the differential effects of 4F-ABUTINACA on adolescent mice and adult mice. Functional enrichment analysis of all differentially expressed genes in hippocampal tissue RNAseq data of adolescent and adult mice after exposure to 4F-ABUTINCA (4 mg/kg, i.p.) or vehicle group **(A,E)**. KEGG pathway or GO term of differentially expressed genes (Padj <0.05) in adolescent and adult mice after exposure to 4F-ABUTINCA (4 mg/kg, i.p.) **(B,F)** or vehicle control **(C–H)** for 2 h. Statistical analyses were performed as described in 2.8. RNA sequencing and bioinformatic analyses are described in the “Materials and methods” section. (Notes on the abbreviations in the figure, Pathways of neuro … diseases: Pathways of neurodegeneration - multiple diseases; Chemical car … oxygen species: Chemical carcinogenesis - reactive oxygen species; Non-alcoh … disease: Non-alcoholic fatty liver disease; Retro … signaling: Retrograde endocannabinoid signaling; Bacterial invas … cells: Bacterial invasion of epithelial cells; Transcriptional mis … in cancer: Transcriptional misregulation in cancer; Parathyroid hormone … and action: Parathyroid hormone synthesis, secretion and action).

**FIGURE 5:**
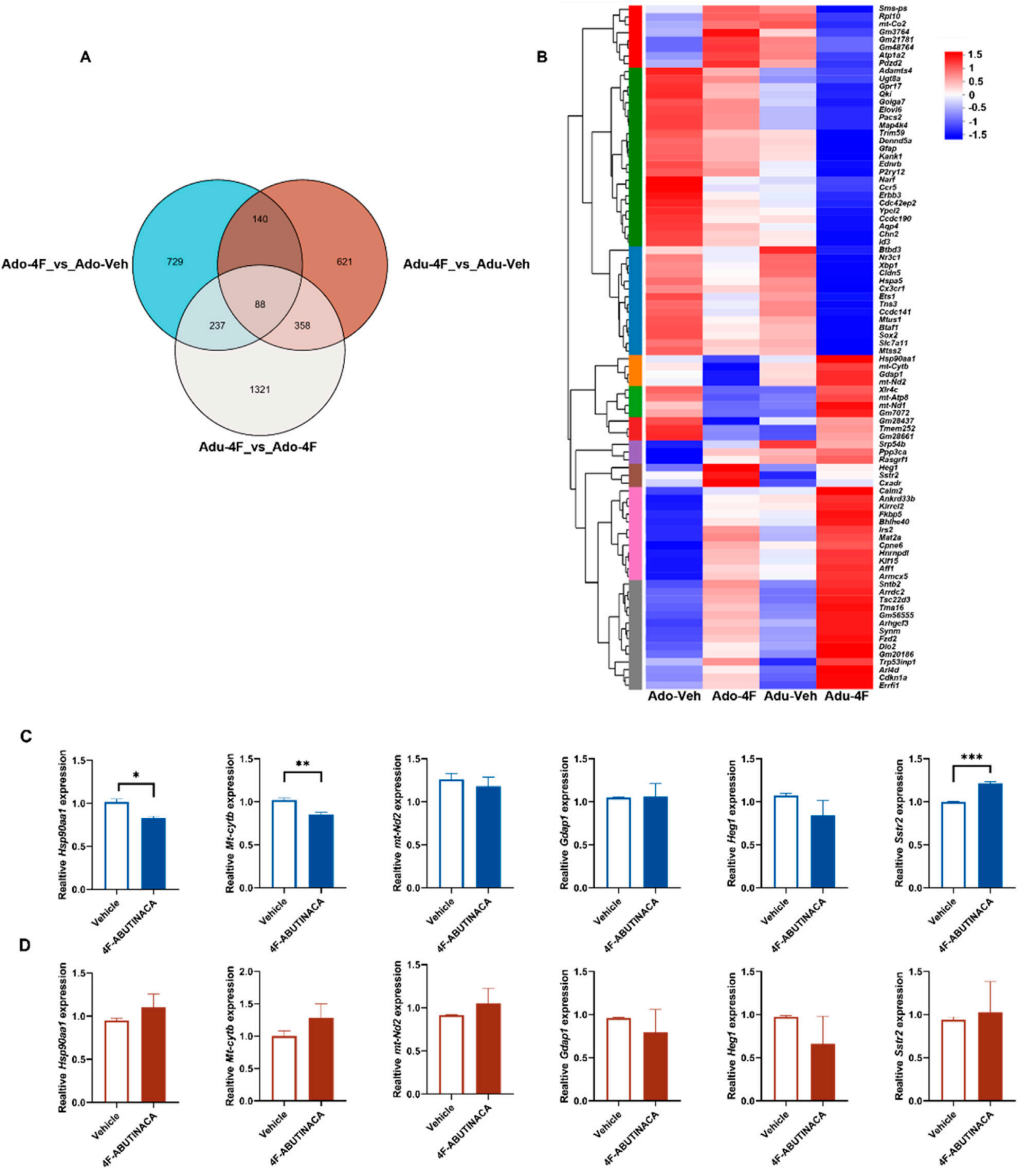
Differential gene expression following 4F-ABUTINACA exposure in adolescent and adult mice. Venn diagram analysis of gene sets in different groups **(A)**. Heatmap showing the effect of transcription of 88 intersecting genes after adolescent, adult 4F-ABUTINACA or vehicle exposure **(B)**. Adolescent **(C)** and adult mice **(D)** were given 4F-ABUTINACA (4 mg/kg, i.p.) or its vehicle, respectively, and their hippocampi were collected 2 h later for qRT-PCR analysis of specific gene transcripts. Statistical analyses were performed as described in 2.8. RNA sequencing and bioinformatic analyses are described in the ‘Materials and methods ‘section. Data are expressed as mean ± SEM. **P < 0.01, ****P < 0.0001 by Student’s t-test (n = 3 per group).

Transcriptomic profiling revealed significant pathway perturbations as demonstrated by KEGG pathway and Gene Ontology (GO) enrichment analyses. Using a threshold of adjusted p-value <0.05 (Benjamini–Hochberg correction), we identified 15 significantly enriched KEGG pathways and 15 GO terms across the three ontological categories (biological process, molecular function, and cellular component) (Figure 4). We started with overall transcriptome analysis of all samples, where the most significantly enriched pathways (Figure 4A) included “Alzheimer’s disease” (Padj 3.72 × 10^−4^), “Parkinson’s disease” (Padj 7.14 × 10^−4^) and “neurodegenerative disease pathways” (Padj 8.11 × 10^−4^). These significantly enriched pathway results reconfirmed that the learning memory function in the hippocampal region of the brain still exists and that the functional role of learning memory cannot be ignored. We then analyzed the differential functional changes of 4F-ABUTINACA exposure in adolescent and adult mice and found that adult and juvenile mice were significantly enriched in Focal adhesion (Padj 7.66 × 10^−13^) and *Salmonella* infection (Padj 3.32 × 10^−10^) (Figure 4B). Notably, many of the Ado-4F mouse gene transcripts were associated with cancer, such as proteoglycans in cancer (Padj 3.14 × 10^−3^) and choline metabolism in cancer (Padj 3.7 × 10^−2^), compared to the vehicle control (Figure 4C). Similar results were found in Adu-4F mice, where transcription of differential genes was significantly enriched in pathways in cancer (Padj 2.89 × 10^−3^), mRNAs in cancer (Padj 3.69 × 10^−3^) and transcriptional misregulation in cancer (Padj 5.17 × 10^−3^) compared to vehicle control (Figure 4D). Transcribed genes from all samples showed a strong enrichment [Response to oxygen-containing compound (GO:1901700, Padj 1.92 × 10^−13^), Response to organic cyclic compound (GO:0014070, Padj 1.17 × 10^−11^) and Regulation of developmental process (GO:0050793, Padj 5.76 × 10^−11^)] in biological processes (BP) (Figure 4E). While adolescent and adult mice exposed to 4F-ABUTINACA (Figure 5F) were dominated by [Biological regulation (GO: 0065007, Padj 3.04 × 10^−111^)] in BP, specific activity in molecular function (MF) had [Protein binding (GO: 0005515, Padj 5.53 × 10^−54^)] and cellular components (CC) highlighted [Cell projection (GO: 0042995, Padj 2.13 × 10^−75^) and Cellular anatomical entity (GO: 0110165, Padj 2.49 × 10^−75^)]. In comparison with the vehicle control, both Ado-4F mice (Figure 4G) and Adu-4F mice (Figure 5H) demonstrated significant enrichment in the categories of Biological Processes (BP) [Biological Regulation (GO: 0065007, Ado-4F mice, Padj 3.09 × 10^−51^; Adu-4F mice, Padj 6.38 × 10^−78^) and Regulation of Biological Process (GO: 0050789,Ado-4F mice, Padj 7.87 × 10^−41^; Adu-4F mice, Padj 1.86 × 10^−74^)] and Molecular Functions (MF) [Protein Binding (GO: 0005515, Ado-4F mice, Padj 1.30 × 10^−44^; Adu-4F mice, Padj 5.53 × 10^−54^) and Binding (GO: 0005488, Ado-4F mice, Padj 2.20 × 10^−27^; Adu-4F mice, Padj 2.97 × 10^−37^)]. Ado-4F mice exhibited marked enrichment in the cellular components (CC), specifically [Membrane-bound organelle (GO: 0043227, Padj 1.98 × 10^−33^)], while Adu-4F mice exhibited [Plasma membrane-bound cell projection (GO: 0120025, Padj 1.99 × 10^−36^)].

### 3.5 Effects of 4F-ABUTINACA on brain hippocampal transcription: differential gene expression

Figure 5A is a Venn analysis plot showing the number of genes in each target gene set and the overlapping relationships between genes in each set. We searched for intersections and found that there were 88 of these genes, which collectively belonged to the Adu-Veh, Adu-4F, Ado-Veh, and Ado-4F groups. We then performed cluster analysis of these 88 genes (Figure 5B) to visualize their expression trends, and combined with the results of the preliminary KEGG pathway and Go term enrichment analyses, we selectively verified the genes that showed large expression differences with Adu-4F and Adopt-4F after administration of 4F-ABUTINACA by q-PCR (heat shock protein 90 alpha family class A member 1 gene, *Hsp90aa1*; mitochondrially encoded cytochrome b gene, *Mt-cytb*; ganglioside induced differentiation associated protein 1, *Gdap1*; mitochondrially encoded NADH dehydrogenase 2 gene, *mt-Nd2*; heart development protein with EGF like domains 1, *Heg1*; and somatostatin receptor 2, *Sstr2*) (Figure 5). The *Hsp90aa1* gene, *Mt-cytb* gene, and *Sstr2* gene were altered in adolescent mice exposed to 4F-ABUTINACA (Figure 5C), whereas there were no statistically significant differences in adult mice (Figure 5D). No significant differences in Gap1 gene, mt-Nd2 gene and Heg1 gene between adolescent and adult mice exposed to 4F-ABUTINACA (Figure 5C; Figure 5D).

Overall, these data highlight behavioral alterations in cognitive function following a single exposure to the synthetic cannabinoid 4F-ABUTINACA, as well as profound age-specific consequences for key molecular components in the hippocampal tissue of the brain.

## 4 Discussion

In this study, we first sought to ascertain whether exposure to the synthetic cannabinoid 4F-ABUTINACA (1, 2, 4 mg/kg) in adolescent mice and young adult mice would result in alterations to their locomotor activity and anxiety-like behavior. We then proceeded to measure the effects on their cognitive functions, with a particular focus on recognition memory capacity, memory extraction capacity, and spatial navigation memory capacity. We found no significant changes in overall locomotor and anxiety-like behaviors in adolescent and adult mice, while the same dose affected recognition memory, memory extraction and spatial navigation memory in adolescent mice and spatial navigation memory in adult mice. The emergence of this interesting result finding in animal behavior was explored in further depth. The hippocampus, a brain region that plays a pivotal role in memory and is characterized by a high density of CB1 receptors, was selected for comprehensive transcription analysis of genes using transcriptomics. This analysis identified differentially expressed genes in adolescent and adult mice. Subsequent functional enrichment analysis revealed pathways associated with cancer and inflammatory factors. qPCR validation further substantiated the key genes implicated in the observed changes in cognitive functions: *Hsp90aa1*, *My-cytb*, and *Sstr2*.

To our knowledge, this study is the first comparative study to assess the effects of synthetic cannabinoid analogues on cognitive function in adolescent and adult mice. However, we were not surprised to find that acute administration of the synthetic cannabinoid analogue 4F-ABUTINACA induced extensive changes in gene expression in hippocampal tissue in the brain. In behavioral studies, where motor ability and anxious behavior were not influences that indirectly caused changes in cognitive functioning in adolescent mice, exposure to 4F-ABUTINACA was found to impair recognition memory, memory extraction and spatial navigational memory, while in adult mice significant differences in spatial navigational memory ability were observed compared to controls. The experimental findings are consistent with other reports indicating that synthetic cannabinoids influence cognitive function; the acute systemic administration of ^Δ^9-THC (8 mg/kg, i.p.) prior to training disrupts memory acquisition in the water maze test without affecting motor performance; this effect is blocked by the CB1 antagonist/reverse antagonist rimonabant (SCIENCE ON, 1982). Positional learning deficits have also been reported in rats treated repeatedly with ^Δ^9-THC(Moore et al., 2010) or acutely with ^Δ^8-THC (Diana et al., 2003) or synthetic CB1 agonists (e.g., HU-210) (Ferrari et al., 1999). Another synthetic cannabinoid, WIN55212-2 (1 and 3 mg/kg), has also been found to impair learning memory in the water maze (Acheson et al., 2011; Tomas-Roig et al., 2017). Furthermore, administration of the synthetic CB1 agonist WIN55212-2 (2.5 mg/kg) 30 min prior to the conditioned reflex phase impaired the acquisition of situational fear conditioning, but not conditioning to independent auditory cues (tones) (Pamplona et al., 2006). Furthermore, systemic or intrahippocampal administration of Δ9-THC or WIN55212-2, either acutely or repeatedly, impaired object recognition in rats (Barna et al., 2007). Suenaga et al. (2008) found that microinjections of WIN55212-2 (1–2 μg/lateral) in the hippocampus 10 min before the first exposure to an object did not affect routine procedures in memory, but impaired the ability to recognize the new space of an object in a CB1-dependent manner. The above reports in the relevant literature on the effects of synthetic cannabinoids on cognitive functioning are only available for experimental populations of a single age group (adults), and there are only a few reports in the current literature on the effects of synthetic cannabinoids on learning memory in adolescent rodents. JACOBS-BRICHFORD E et al. reported that using an attentional stereotyped shifting task and a probabilistic rewarded choice task, adolescent male and female rats administered WIN 55,212 - 2 showed persistent subtle deficits in cognitive processes related to flexibility and decision-making (Jacobs-Brichford et al., 2019).

The hippocampus is also an important part of the limbic system of the human brain, which plays an important role in spatial navigation and the consolidation of information from short-term to long-term memory (Zhong et al., 2020). There is a high density of CB1 cannabinoid receptors in the hippocampus (Herkenham et al., 1990) and direct injection of cannabinoids into this region impairs memory (Lichtman et al., 1995). We further extracted hippocampal tissues exposed to 4F-ABUTINACA at different ages separately and assessed its effect on gene transcription levels. 4F-ABUTINACA triggers alterations in a variety of signaling pathways including, but not limited to, Alzheimer’s disease, Parkinson’s disease and neurodegenerative diseases. Significant differences in the rates of focal adhesion, *Salmonella* infection, and axonal phagocytosis were observed between adolescent and adult mice exposed to 4F-ABUTINACA. In contrast, the activated endocannabinoids system (eCBs) affects fibroblast remodeling by reducing metalloproteinases secreted by fibroblasts through lipid rafts associated with focal adhesions (McPartland, 2008). Interestingly, the natural cannabinoid ^Δ^9-THC does not cause induction of his^+^ revertants in *Salmonella* tester strains (Ferk et al., 2016), but extensive changes were observed in our experiments, which may be the result of synthetic cannabinoids have higher toxicity (Luo, 2025). Differences in developmental changes in the adolescent adult brain are also another proof that led to the results of this experimental study. Mulder et al. (2008) found that eCBs have recently been identified as axon guidance cues shaping the connectivity of local GABAergic interneurons in the developing cerebrum, they showed that eCB signaling is operational in subcortical proliferative zones from embryonic day 12 in the mouse telencephalon and controls the proliferation of pyramidal cell progenitors and radial migration of immature pyramidal cells. When layer patterning is accomplished, developing pyramidal cells rely on eCB signaling to initiate the elongation and fasciculation of their long-range axons. The activation of the Axon- Guidance signaling system, which leads to the upregulation of the *Sstr2* gene (Smit-McBride et al., 2011), was also confirmed, which at the same time provides evidentiary support for the key target genes identified in our study as causing cognitive differences between adolescent and adult mice after exposure to 4F-ABUTINCA. *Mt-Cytb* is a component of the ubiquinol-cytochrome c reductase complex (complex III or cytochrome b-c1 complex) that is a part of the mitochondrial respiratory chain. Altered expression has been shown to correlate with mitochondrial homeostasis in neurodegenerative diseases (Czapski et al., 2018), and also as a neuroprotective target with altered mRNA levels of mitochondria-associated proteins in animal models of Alzheimer’s disease (Żulińska et al., 2024), and recent studies have identified that altered expression of the *Mt - Cytb* gene, which regulates the mitochondrial respiratory chain, impairs early development in mouse oocytes (Cheng et al., 2022). *Hsp90aa1* is a molecular chaperone that promotes the maturation, structural maintenance, and proper regulation of specific target proteins involved in cell cycle control and signal transduction among other processes, and is a biomarker for the stress response. *Hsp90aa1* may be upregulated in the hippocampus after maternal deprivation as part of a compensatory response to mitigate injury. Early maternal deprivation impairs learning memory and alters *Hsp90aa1* gene expression in the hippocampus of adult male rats reveals that *Hsp90aa1* may be a promising therapeutic target for treating chronic stress-induced hippocampal damage and spatial learning and memory dysfunction (Xiong et al., 2021). Our experiment adds another strong support to reveal that *Hsp90aa1* may be a key target for dominating hippocampal spatial learning and memory function.

Our study reveals for the first time the behavioral effects on cognitive function and associated molecular changes in adolescent and adult male mice after single exposure to the synthetic cannabinoid 4F-ABUTINACA, filling a gap in this regard and providing compelling evidence for subsequent studies of targeted therapies in specific groups. However, it should be noted that there are some limitations in this study. Milene Borsoi et al. report that Prefrontal cortex (PFC) neuronal and synaptic function is differentially affected by a single exposure to the synthetic cannabinoid mimetic WIN55,212–2 for 24 h in male and female rats during adolescence and adulthood. During adolescence, single cannabinoid exposure (SCE) reduced play behavior in female but not male rats. In contrast, the same treatment impairs sociability in adult male and female rats. General exploration and memory recognition remained normal at both ages and in both sexes. At the synaptic level, SCE abrogated endogenous cannabinoid-mediated synaptic plasticity in the PFC of females of both ages and increased excitability of PFC pyramidal neurons in adulthood, whereas males were spared. In contrast, cannabinoid exposure was associated with impaired long-term potentiation (LTP) in adult males (Borsoi et al., 2019). Cristina Izquierdo-Luengo et al. found reduced prepulse inhibition of the startle reflex was found in male (but not female) mice in mice exposed to the short- and long-term effects of JWH-018 sensory-motor gating. Furthermore, adolescent exposure to JWH-018 induced an activation of microglia and astrocytes in the prefrontal cortex of male mice at both time intervals. A transitory decrease in the expression of GAD67 and CB2 cannabinoid receptors in the prefrontal cortex was also found in male mice exposed to JWH-018 (Izquierdo-Luengo et al., 2023). In another study, Higuera-Matas et al. (2012) found that repeated administration of the cannabinoid agonist CP 55,940 to adolescent rats disrupted the normal balance between glutamate and GABA transmission to a greater extent in females than in males. The exact reason for this is not clear. One possible explanation is that the effects of cannabinoids in females may be subject to dynamic changes as a function of fluctuating gonadal sex hormones (i.e., estrogen and progesterone) (Fogel et al., 2017). In future experiments, we will pay more attention to the gender factor. It would also be beneficial to further examine the variable of gender factors in future studies.

## 5 Conclusion

This study demonstrated that a single acute exposure to the synthetic cannabinoid 4F-ABUTINACA induced behavioral changes in cognitive function in addition to locomotor and anxiety-like behaviors, as well as extensive and complex alterations in the hippocampal transcriptome of the mouse brain, which were differentially affected by age-specific effects, in adolescent and adult mice. In adolescent mice, 4F-ABUTINACA altered the expression of related genes. Future studies will need to address the functional significance of the targeting and the molecular mechanisms involved for specific age groups.

## Data Availability

The original contributions presented in the study are publicly available. This data can be found here: NCBI repository, Accession: PRJNA1278941 (https://www.ncbi.nlm.nih.gov/biosample/49325492, https://www.ncbi.nlm.nih.gov/biosample/49325493, https://www.ncbi.nlm.nih.gov/biosample/49325494, https://www.ncbi.nlm.nih.gov/biosample/49325495, https://www.ncbi.nlm.nih.gov/biosample/49325496, https://www.ncbi.nlm.nih.gov/biosample/49325497, https://www.ncbi.nlm.nih.gov/biosample/49325498, https://www.ncbi.nlm.nih.gov/biosample/49325499 https://www.ncbi.nlm.nih.gov/biosample/49325500, https://www.ncbi.nlm.nih.gov/biosample/49325501 https://www.ncbi.nlm.nih.gov/biosample/49325502, https://www.ncbi.nlm.nih.gov/biosample/49325503).
