# Supplementary material for: Age-specific effects of synthetic cannabinoids on cognitive function and hippocampal gene expression in mice: insights from behavioral and molecular correlates
Source: Front Pharmacol. 2025 Jun 20;16:1618929. doi: 10.3389/fphar.2025.1618929 (PMC12231510; doi:10.3389/fphar.2025.1618929)
Supplement: Supplementary file 1 [file DataSheet1.PDF]

Supplementary Material

Primer design sequence:

Hsp90aa1: forward, 5′-A CTCCAATTCATCGGACGCTCT -3′;
reverse, 5′- GTCAGGGTTCGGTCCTGTTT -3′;

Mt-Cytb: forward, 5′- GGACGAGGCTTATATTATGGA -3′;
reverse, 5′- AATGCTGTGGCTATGACTG -3′;

Gdap1: forward, 5′- CACCCAGGTTAATGCCCGAT -3′;
reverse, 5′- GTGTTACCAATCTGGCTGCG -3′;

mt-Nd2: forward, 5′- ACAAGCAACAGCCTCAAT -3′;
reverse, 5′- GTGTTACCAATCTGGCTGCG -3′;

Heg1: forward, 5′- GAGCGCGGCCTTGGAG -3′;
reverse, 5′- GCCTAGGGATGTCCCACTCT -3′;

Sstr2: forward, 5′- GCTCGAGGAAAACCAAGATGT -3′;
reverse, 5′- CTGGTTGGAGCCATTGCTTG -3′;

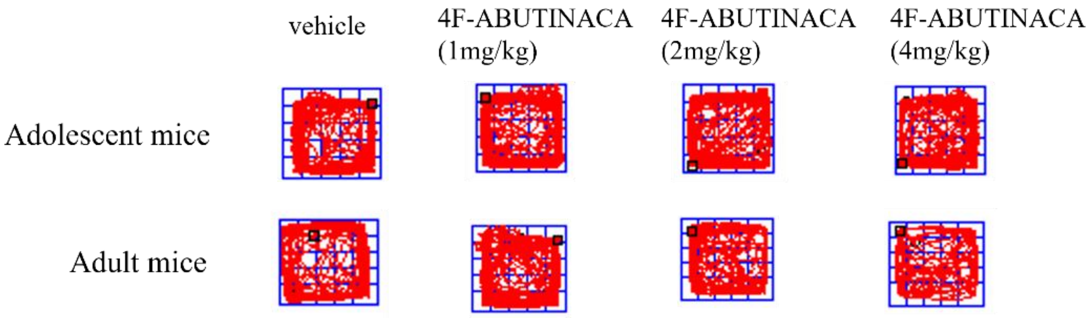

Fig. S1. Locomotor activity mice trajectory plot.

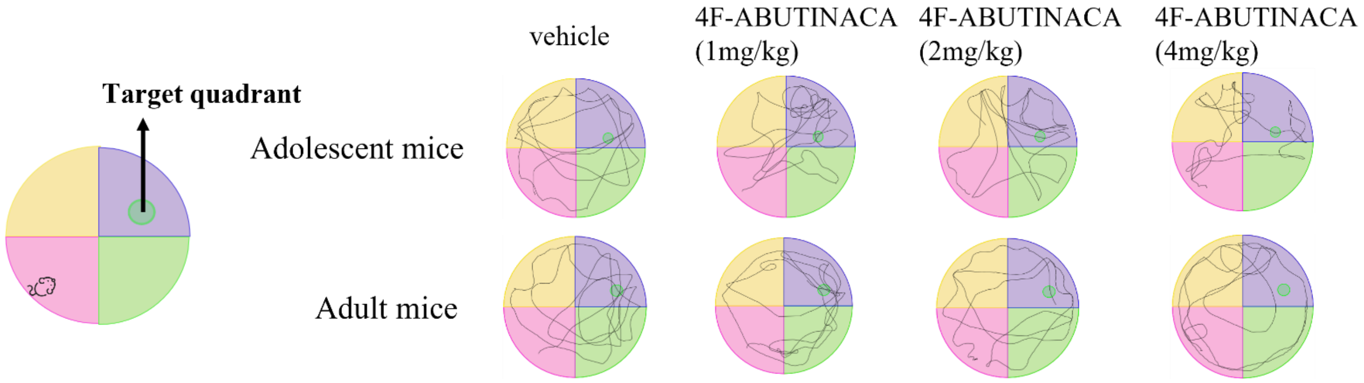

Fig. S2. Morris Water Maze mice trajectory plot.
